# Protective effects of mung bean (*Vigna radiata* L.) and pea (*Pisum sativum* L.) against high‐fat‐induced oxidative stress

**DOI:** 10.1002/fsn3.1271

**Published:** 2019-11-21

**Authors:** Dandan Liu, Xiao Guan, Kai Huang, Sen Li, Jing Liu, Wenwen Yu, Ruiqian Duan

**Affiliations:** ^1^ School of Medical Instruments and Food Engineering University of Shanghai for Science and Technology Shanghai China; ^2^ College of Information Engineering Shanghai Maritime University Shanghai China

**Keywords:** antioxidant capacity, ethanol extracts, mung beans, peas

## Abstract

Hyperlipidemia is closely related to oxidative stress, and it has been proved that the intake of legumes can protect the body from chronic diseases related to oxidative stress. In this study, we investigated the protective effects of mung beans and peas against high‐fat‐diet‐induced rats. It was found that, with 50% addition of mung beans or peas, the intake of mung beans and peas could significantly restore the levels of serum total cholesterol, low‐density lipoprotein cholesterol, and high‐density lipoprotein cholesterol. Liver staining also showed that high‐fat diet (HFD) led to liver lesions, whereas whole‐grain intake could significantly relieve these symptoms. Compared with the HFD group, the antioxidant defense system and antioxidant gene expression in administered legume groups improved markedly. Furthermore, the antioxidant activities of the two legume extracts were determined. Characterization showed that the ethanol extracts of mung beans and peas possessed high antioxidant activities, for their ability to scavenge ABTS and DPPH, reduce Fe^3+^ and their antilipid peroxidation capacity. Treatments with ethanol extracts at different doses could restore the levels of intracellular lipid, malondialdehyde, and antioxidant enzyme activities in oleic acid‐induced HepG2 cells. All these results suggested that mung beans and peas or their extracts may be utilized as good candidates of natural antioxidant agents.

## Graphical abstract

Intake of mung bean and pea could dramatically alleviate the serum and liver lipid profile of the high‐fat‐diet‐induced rats and markedly improved the antioxidant defense system and antioxidant gene expression. Characterization showed that the ethanol extracts of mung beans and peas possessed high antioxidant activities in vitro. Treatments with ethanol extracts at different doses could restore the levels of intracellular lipid, malondialdehyde, and antioxidant enzyme activities in oleic acid‐induced HepG2 cells.The main contribution of this paper is to validate the antioxidant capacity of mung beans and peas by animal experiments in vivo, chemical experiments in vitro, and HepG2 cell antioxidant experiments. In addition, the interaction between legumes and the rats fed with HFD was studied by HE staining and gene expression.

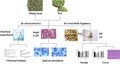

## 1 INTRODUCTION

With the improvement of people's quality of life and the adjustment of dietary structure, hyperlipidemia has increasingly become one of the chronic diseases affecting human health (Ramalingam & Kim, 2014). It is characterized by elevated serum total cholesterol (TC) and low‐density lipoprotein (LDL) and very LDL cholesterol and decreased high‐density lipoprotein (HDL) levels (Assmann & Gotto, 2004). Lipid metabolism disorder and lipid peroxidation injury caused by hyperlipidemia are the main factors for cardiovascular and cerebrovascular diseases (Kristensen et al., 2012). Some studies have proved that hyperlipidemia is closely related to oxidative stress. Hyperlipidemia is an important state that promotes the accumulation of reactive oxygen species (ROS) through several metabolic pathways (Nwosea, Jelineka, Richardsa, Tinleya, & Kerrb, 2009). The imbalance between the oxidative and antioxidant states of human body leads to excessive free radicals, which are harm to the body (Costa et al., 2015).

There are several mechanisms in human body for dietary antioxidants to protect against ROS. Increased intake of antioxidants could have the health effects such as reducing the incidence of cancer and cardiovascular diseases. Legume plays an important role in providing nutrients to people around the world (Summo, Centomani, Paradiso, Caponio, & Pasqualone, 2016). Consumption of legumes is correlated to a number of positive health benefits. These benefits are known to be associated with phytochemicals present in legumes (Magalhaes et al., 2017). Significant antioxidant activities and phenolic compounds in several consumed legumes have been reported, and a legume‐based dietary can decrease the risk of oxidative stress‐related chronic diseases (Liu, Liu, Chen, Chang, & Chen, 2013). Phenolic compounds including phenolic acids, flavones, isoflavones, and condensed tannins have been identified and characterized in food legumes (Cheng, 2009).

Mung bean (*Vigna radiata L.*) is one of the main leguminous plants rich in proteins, vitamins, and minerals (Mubarak, 2005). Mung bean is also abundant in bioactive substances (Mattila, Pihlava, & Hellstrom, 2005), such as flavonoids, alkaloids, and tannins. Flavonoids with antioxidant activity are one of the most studied compounds in recent years for their antioxidant, anticancer, antibacterial, anti‐inflammatory, and hypolipidemic effects (Randhir & Shetty, 2007). Although many studies have been carried out to investigate the functional components and functions of mung beans at home and abroad in vitro, experimental data in vivo are still lacking.

Pea (*Pisum sativum L.*) has a nutritionally favorable composition with respect to macronutrients including low‐fat‐rich protein and high fiber. Moreover, peas have high contents of antioxidant components mainly include phenols, tocopherols, and carotenoids. Duenas, Estrella, and Hernandez (2004) found that peas contain a variety of phenolic compounds, especially in the seed coat. Some studies have shown that other antioxidants are present in peas, however, some of which are poorly characterized. The concentration of polyphenols in peas varied associated with the processing treatments.

Previous studies were mostly focused on the in vitro antioxidant effects of polyphenols in legumes, whereas few studies investigated the effects of intact legumes on antioxidant enzyme activity in vivo and legume extracts on oxidative stress in cells. Therefore, in this study, we assessed and compared the influence of intact mung beans and peas on the antioxidant defense system of rats induced with high‐fat diet (HFD). The antioxidant components of mung bean and pea extracts were further validated in vitro or performed with HepG2 cell antioxidant experiments.

## 2 MATERIALS AND METHODS

### 2.1 Chemicals and reagents

The mung beans and peas were obtained from a food company in Shanxi Province, China. Kits for catalase (CAT), TC, triglyceride (TG), low‐density lipoprotein cholesterol (LDL‐C), total antioxidant capacity (T‐AOC), and high‐density lipoprotein cholesterol (HDL‐C) were purchased from Jiancheng Bioengineering Institute, Nanjing, China. Kits for malondialdehyde (MDA), total superoxide dismutase (SOD), glutathione peroxidase (GSH‐Px), MTT Cell Proliferation and Cytotoxicity Assay Kit (MTT), Reactive Oxygen Species Assay Kit (ROS), TRIzol, and cDNA synthesis were purchased from Beyotime Biotechnology. Oil Red O was obtained from Sigma‐Aldrich. Potassium ferricyanide, trichloroacetic acid, thiobarbituric acid, 2,2′‐azino‐bis (3‐ethylbenzothiazoline‐6‐sulfonic acid) diammonium salt (ABTS), and 2,2‐diphenyl‐1‐picrylhydrazyl radical (DPPH) were purchased from Vita Co. Ltd. All chemicals and solvents were of corresponding grade required for the experiment.

### 2.2 Extract preparation

Mung beans and peas were milled into flour and thoroughly mixed, and then stored at 4°C for further analysis. Nutritional components of mung beans and peas were provided by China Agricultural University (Table 1). A portion of the processed samples were obtained for sequential extraction, and the rest was added into animal feeds.

**Table 1:** The component contents of mung bean and pea

Component content	Mung bean	Pea
Energy (KJ/100 g)	1,397	1,451
Protein (g/100 g)	24.5	23.7
Fat (g/100 g)	1.5	1.4
Carbohydrates (g/100 g)	49.8	55.5
Total dietary fiber (g/100 g)	9.74	6.61
Ash content (g/100 g)	2.3	2.8
Moisture (g/100 g)	12.2	9.96
Amino acids (g/100 g)	22.45	21.2
Vitamin E (mg/100 g)	8.9	12.3
Phosphorus (mg/100 g)	322	349
Potassium (g/kg)	8.52	9.19
Manganese (mg/100 g)	10.4	12.6
Selenium (mg/100 g)	＜0.01	0.0286
Quercetin (μg/100 g)	＜0.1	＜0.1
Ferulic acid (mg/100 g)	17.6	2.33
Protocatechuic acid (mg/100 g)	400	11.1
Vanillic acid (mg/100 g)	12.2	14.4

The extraction method was carried out based on a previously described method with slight modifications (Faller, Fialho, & Liu, 2012). Briefly, the flours were placed in a shaking water bath with n‐hexane (1:4 ratio), which was used to extract lipids from the samples, at 58°C for 2 hr. After the lipids were removed, the grain flours were dried and extracted with deionized water (1:10 ratio) at 50°C for 2 hr and centrifuged at 4,500 r/min for 20 min. Supernatants were regarded as the water extracts, and the precipitates were freeze‐dried. Then, the water‐insoluble fractions were mixed with 80% ethanol (1:10 ratio) at 60°C for 2 hr and then centrifuged. The precipitates were discarded, and ethanol was evaporated by using a rotary evaporator. The ethanol extracts were obtained after freeze‐dried.

### 2.3 Antioxidant activity

#### 2.3.1 Animal experiments

A total of 48 male Sprague‐Dawley rats (200 ± 5 g) were donated by Shanghai Slac Laboratory Animal Co. Ltd. The license number for using experimental animals was SCXK(SH)2017‐0005. All the rats were maintained under standard conditions (22°C ± 2°C, relative humidity 55% ± 0.5%, 12‐hr light–dark cycle). Surgery was performed under anesthesia to minimize suffering.

After one‐week acclimatization phase, the rats were randomly separated into six groups (eight rats each): normal fat diet (NFD) group, high‐fat diet (HFD) group, HFD with 10% mung bean (HFDM‐L) group, high‐fat diet with 50% mung bean (HFDM‐H) group, high‐fat diet with 10% pea (HFDP‐L) group, and high‐fat diet with 50% pea (HFDP‐H) group. Feed was purchased from Jiangsu Xietong Pharmaceutical Bioengineering Co., Ltd. HFD included 15% lard, 10% sucrose, 1.2% cholesterol, 0.2% cholic acid, and basal diet. 10% and 50% (w/w) mung bean and pea flours were added into the HFD resulting in HFDM‐L, HFDP‐L, HFDM‐H, and HFDP‐H, respectively. The rats of each group were fed with the corresponding diet for 4 weeks. The body weight was measured every week. At the end of this experiment, the rats were deprived of food overnight and sacrificed. For the serum preparation, the rats were anesthetized, and blood samples were collected from the abdominal aorta. The livers were immediately dissected, weighed, and stored at −80°C for analysis.

#### 2.3.2 Biochemical analysis

##### Analysis of serum biochemical values

TG, TC, LDL‐C, and HDL‐C levels in the serum were analyzed using a Multi‐Mode Microplate Reader (Synergy HTX systems; Biotex) according to the manufacturer's instructions.

##### Assessment of SOD, MDA, GSH‐Px, CAT, and T‐AOC

The activities of SOD, MDA, GSH‐Px, CAT, and T‐AOC were tested in line with the kit's instructions. Liver homogenates were prepared in an ice bath with tissue homogenizers to determine the liver antioxidant enzyme activities, and the supernatants were obtained via centrifugation at 12,000 r/min for 10 min.

#### 2.3.3 Histopathological investigations

The livers were collected, fixed, stained with hematoxylin and eosin, and observed under a light microscope (Shen et al., 2017).

#### 2.3.4 Gene expression in the liver

Firstly, the total RNA in rat liver was first extracted by TRIzol Kit following the manufacturer's instructions. Then, the total RNA was reverse‐transcribed into cDNA. Real‐time polymerase chain reaction was used to quantify the expression of mRNA, and the primers are listed in Table 2.

**Table 2:** Sequences of primers used in quantitative real‐time reverse transcription PCR

Gene	Forward primer	Reverse primer
Nrf2	CCCAGCACATCCAGAC	GGGCAAGCGACTCAT
NQO1	ATGGCGGTGAGAAGAGC	AAGTTCATAGCATAGAGGTCAGATT
CAT	CGCCATTGCCACAGGA	GATGAGAGGGTAGTCCTTGTG
SOD	GCGTGCTGAAGGGCGA	CCTGCTGTATTATCTCCAAA
HO‐1	ACCAAGGACCAGAGCCCC	GTAAGGACCCATCGGAGAA

### 2.4 Antioxidant activity in vitro

Because antioxidants have various mechanisms in the oxidation–reduction process, the use of a variety of assays to determine antioxidant activity may help us in better understanding these mechanisms.

#### 2.4.1 Radical scavenging activity

##### Free radical scavenging activity on DPPH

The antioxidant potential of the obtained extracts was tested by using DPPH assay as described by Shen et al. (2017) with slight modifications.

##### Free radical scavenging ability on ABTS

The ABTS scavenging assay was performed following the method described by Zhao et al. (2006) with some modifications.

#### 2.4.2 Reducing power

The reducing power of the extracts was determined by using the assay described by Bamdad, Wu, and Chen (2011). Phosphate‐buffered saline (PBS) was used as a blank reagent.

#### 2.4.3 Antilipid peroxidation capacity

The antilipid peroxidation capacity was assessed using the procedures described by Liu and Huang (2018) with some modifications. PBS was used as a blank reagent.

#### 2.4.4 Determination of total phenolic content

Total phenolic content (TPC) in mung bean and pea was determined by a Folin–Ciocalteu assay with slight modifications (Guo, Li, Tang, & Liu, 2012) using gallic acid as the standard. The TPC was expressed as milligram gallic acid equivalents per gram dry legume (mg GAE/g) through the calibration curve of gallic acid.

### 2.5 Antioxidant activity in HepG2 cells

#### 2.5.1 Cell culture and treatment

The cell culture was following by Faller et al. (2012) with slight modifications. OA was dissolved in 1% bovine serum albumin, and an OA‐inducing medium with 1 mM was obtained. The final concentration of ethanol extracts was 100, 200, and 400 μg/ml by adding extracts and OA‐inducing medium into cell culture medium as a treatment group for 12 hr.

#### 2.5.2 MTT assay experiment

Cell viability was tested in line with the MTT Cell Proliferation and Cytotoxicity Assay Kit's instructions.

#### 2.5.3 Oil Red O staining and biochemical analysis in cells

After treatment with the extracts, lipid droplet accumulation cells were measured with Oil Red O staining (Xiao et al., 2016). The stained photographs were taken with a microscope (Olympus, Japan).

The TG and TC accumulated in cells were measured by a Multi‐Mode Microplate Reader. The level of ROS in the HepG2 cells was detected using ROS Assay Kit. The fluorescence intensity of each well was measured immediately at Ex/Em = 488/525 nm by Multi‐Mode Microplate Reader (Tavsan & Kayali, 2019). The levels of SOD, MDA, and GSH‐Px in cells were determined according to manufacturer's instructions.

### 2.6 Statistical analysis

At least three independent trials were conducted in all experiments, and the data were expressed as mean ± standard deviation. The results were analyzed for variance using the GraphPad Prism software (version 6.01; GraphPad Inc.), and statistical significance of differences (*p* < .05) was evaluated using Dunnett's multiple comparisons test.

## 3 RESULTS

### 3.1 Effects of legumes on the growth of rats

Table 3 shows the growth changes in experimental rats after being fed with different diets for 4 weeks. In the present study, the final body weight and liver weight of rats fed with HFD were 12.26% and 70.71% higher than those rats fed with NFD (*p* < .05), and there was no significant difference between legume supplementation groups and HFD group. Compared with the HFD group, the liver index of rats in NFD, HFDM‐L, and HFDM‐L groups decreased significantly (*p* < .05). In addition, there were no significant changes in the food intake of each experimental group (*p* > .05), indicating that different diets had no significant effect on the food intake of rats.

**Table 3:** Effects of cooked mung bean and cooked pea supplementation for 4 weeks on body weights, liver weight, liver index, and food intake in high‐fat diet rats (*n* = 8)

Group	Initial body weight (g)	Final body weight (g)	Liver weight (g)	Liver index (%)	Food intake (g/day)
NFD	221.44 ± 2.43^a^	394.19 ± 6.98^a^	12.12 ± 0.58^a^	0.03 ± 0.001^a^	25.27 ± 1.25^a^
HFD	218.39 ± 3.35^a^	442.52 ± 12.65^b^	20.69 ± 1.11^b,c^	0.05 ± 0.002^b^	23.58 ± 3.52^a^
HFDM‐L	222.70 ± 2.73^a^	441.62 ± 12.11^b^	19.38 ± 0.69^b^	0.04 ± 0.001^c^	24.58 ± 1.52^a^
HFDP‐L	220.34 ± 2.98^a^	443.44 ± 9.27^b^	22.05 ± 0.80^c^	0.05 ± 0.002^b^	25.14 ± 1.66^a^
HFDM‐H	219.56 ± 2.87^a^	433.15 ± 8.73^b^	18.71 ± 0.56^b^	0.04 ± 0.001^c^	24.97 ± 1.58^a^
HFDP‐H	221.23 ± 3.06^a^	434.35 ± 12.02^b^	20.31 ± 1.76^b,c^	0.05 ± 0.002^b^	24.28 ± 1.26^a^

Note

Data are represented as mean ± SE. ANOVA analysis: Within each row, means with different superscript (a, b, c) are significantly different at *p* < .05, whereas mean superscripts with the same letters mean that there is no significant difference at *p* > .05.

Liver index (%) = (liver weight/body weight) × 100.

### 3.2 Effects of legumes on serum lipid profile

**Figure 1:**
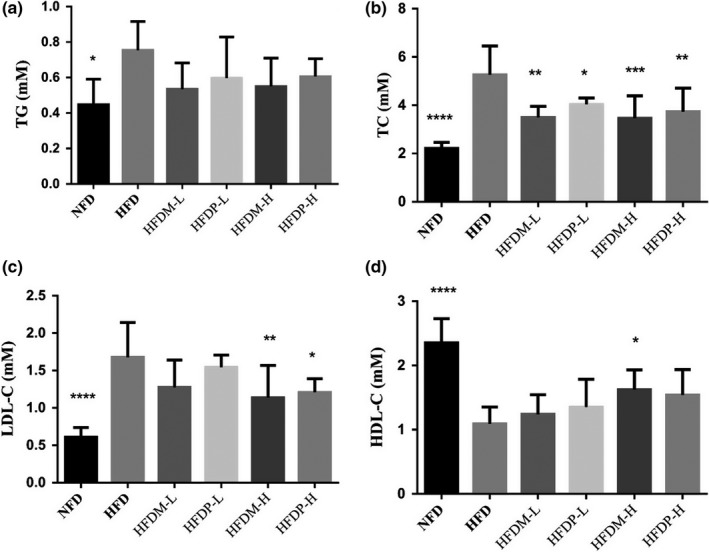
Effects of mung beans and peas for 4 weeks on serum lipids in hyperlipidemia rats. Results are expressed as mean ± *SD* for eight independent experiments (animals) performed in duplicate. ***p < .001, **p < .01, and *p < .05 versus HFD

As shown in Figure 1(a–d), dyslipidemia in HFD rats was observed. Compared with the NFD group, the serum levels of TC, TG, and LDL‐C in the HFD group increased markedly, and the HDL‐C level decreased significantly. Compared with HFD rats, the TC levels in HFDM‐L and HFDP‐L rats increased by 33.57% and 23.37%, respectively. The HDL‐C and TC levels of rats, fed with HFDM‐H, were 34.19% and 48.57% higher than those of HFD rats. Moreover, the LDL‐C level was reduced by 32.28% (*p* < .05). Treatments with mung beans and peas revealed significant decline in serum lipid profile in a dose‐dependent manner. With a higher dose, the levels of TC and LDL‐C did go lower, and bigger of the HDL‐C level. Interestingly, a higher dose of legumes did not have an effect on the levels of TG, though they were lower than the HFD ones.

### 3.3 Effects of legumes on liver lipid profile

**Figure 2:**
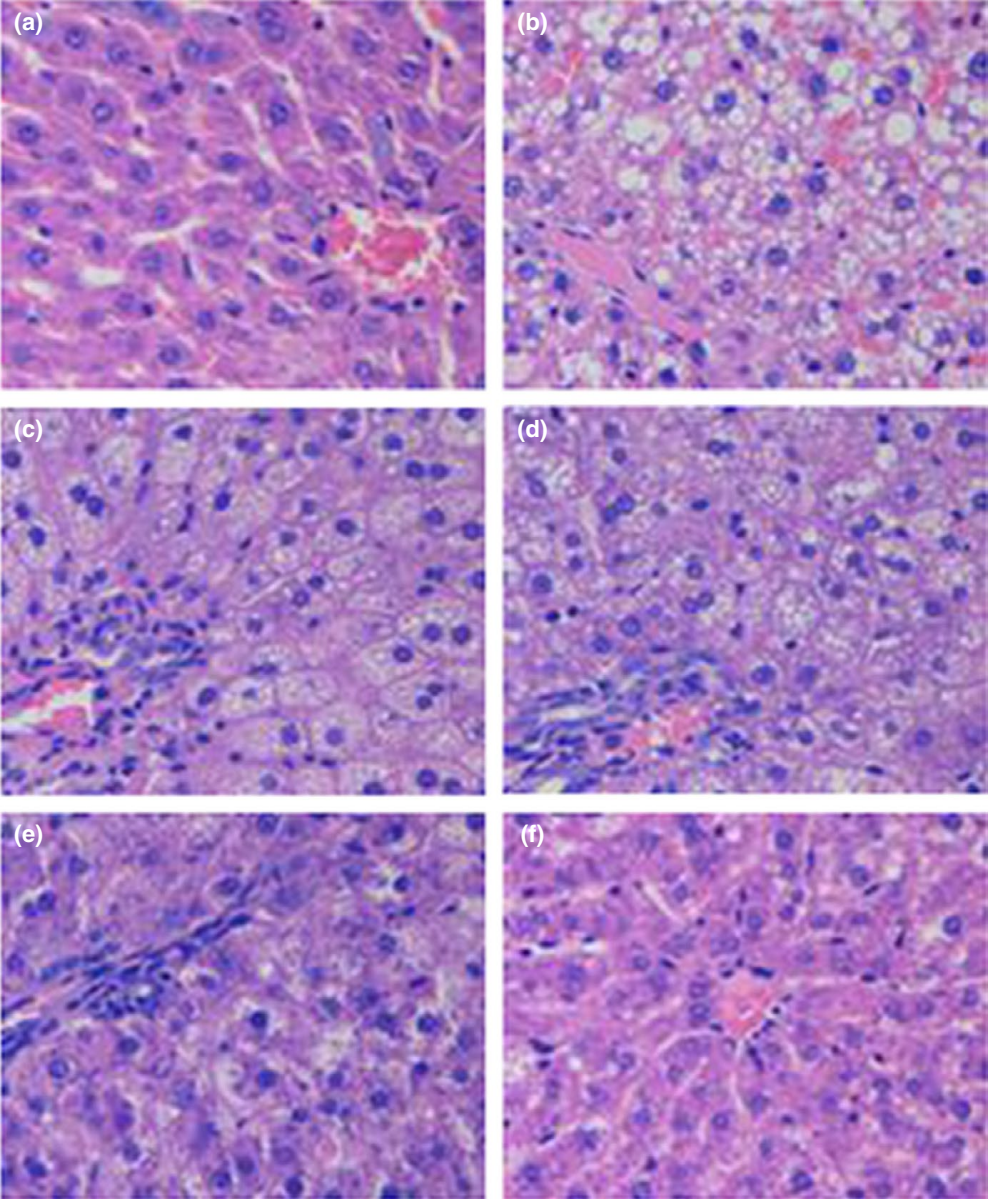
Histopathology of liver (HE, 400×). (a) NFD; (b) HFD; (c) HFDM‐L; (d) HFDP‐L; (e) HFDM‐H; and (f) HFDP‐H

H&E sections of rat livers in each group (Figure 2a–f) showed that the hepatocytes in the NFD rats retained normal morphology including central veins, clear outline of hepatic impellers, and almost no fat droplets (Figure 2a). However, in the HDF rats, the structure of hepatic lobules was disordered, the number of lipid droplets in the cells increased, the volume of lipid droplets became bigger, and a small number of hepatocytes had steatosis. Compared with the HDF group, the lipid droplets in the hepatocytes of all legume groups were significantly reduced, and their volumes were decreased. All these above indicated that mung bean and pea had certain effects in assisting the prevention of fatty liver.

### 3.4 Effects of legumes on antioxidant status

**Figure 3:**
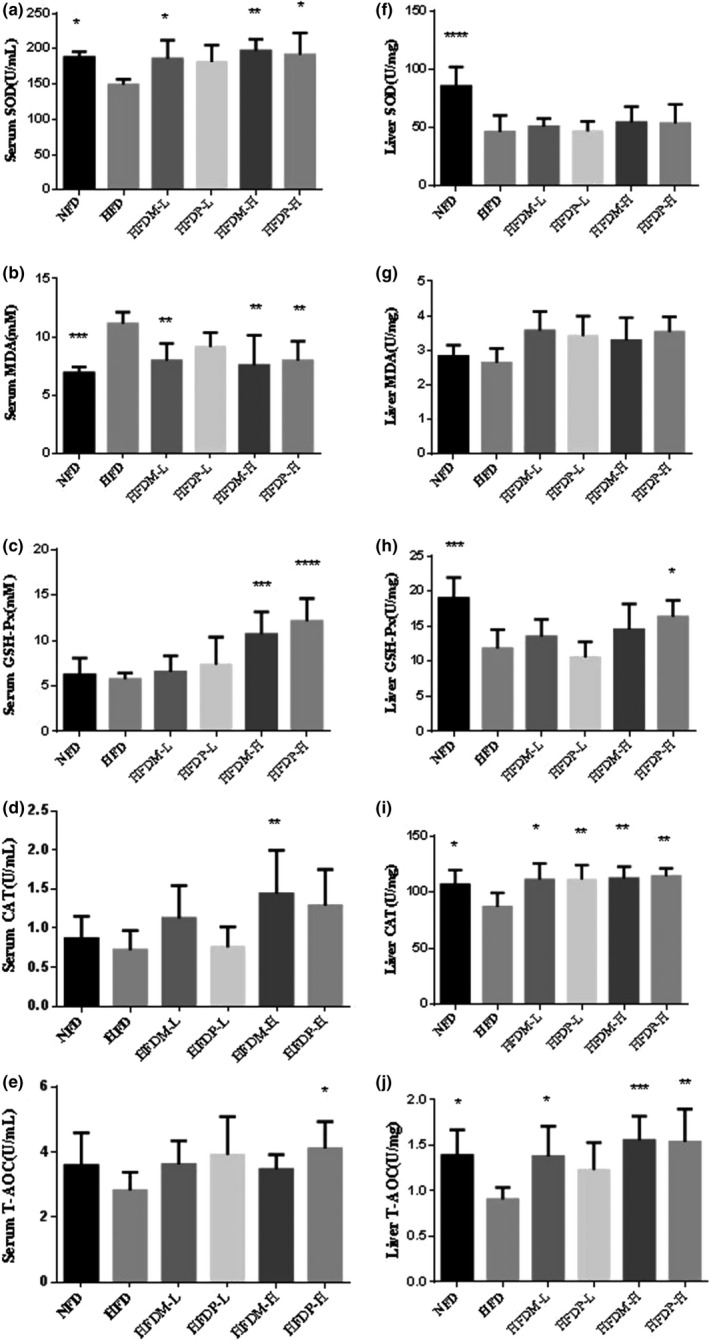
Effects of mung beans and peas for 4 weeks on serum (a–e) and liver tissue (f–j) antioxidant enzymes in hyperlipidemia rats. Results are expressed as mean ± *SD* for eight independent experiments (animals) performed in duplicate. ***p < .001, **p < .01, and *p < .05 versus HFD

Lipid peroxidation and antioxidant enzymes both in serum and in liver were determined (shown in Figure 3). It was found that high‐fat diet would disorder the levels of MDA and the activities of the antioxidant enzymes. However, the intake of mung beans and peas could relieve these symptoms. Compared with the HFD rats, administration of legumes at a higher content could significantly increase the enzyme activities and decrease the level of MDA. In HFDM‐H rats, the activities of serum SOD, CAT, and GSH‐Px were increased by 32.09%, 86.13%, and 100.40%, respectively, and serum MDA level reduced by 31.86% (*p* < .05). In HFDP‐H rats, the activities of serum GSH‐Px, SOD, and T‐AOC increased by 110.99%, 28.56%, and 45.64%, respectively, and serum MDA level reduced by 28.44%. Different from the data of serum enzymes and MDA, the effects on antioxidant status in livers were observed mainly in liver CAT and T‐AOC levels, which were increased by 28.82% and 71.45% for HFDM‐H rats and 31.27% and 69.41% for HFDP‐H rats, respectively.

**Figure 4:**
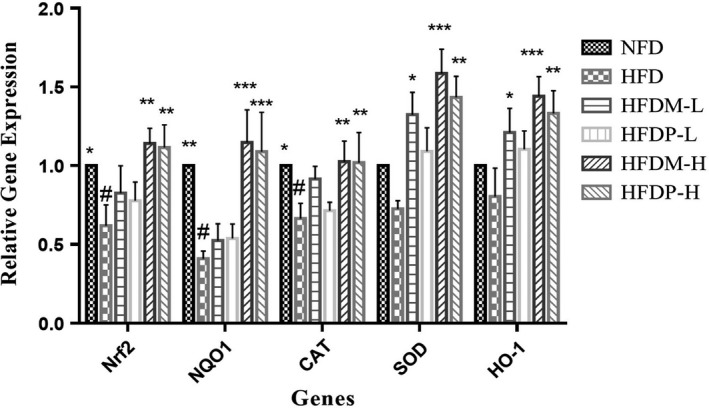
Effects of mung beans and peas on antioxidant gene expression in liver. #*p* < .05 versus NFD group, and *p < .05, **p < .01, and ***p < .001 versus HFD group

As shown in Figure 4, the relative gene expression levels of CAT, SOD, heme oxygenase 1 (HO‐1), transcription factor NF‐E2‐related factor 2 (Nrf2), and NAD(P)H quinone dehydrogenase 1 (NQO1) in HFDM‐L and HFDP‐L rats were markedly increased by a higher dose legume diet compared with the HFD group. In rats fed with diets containing 10% mung bean, the mRNA levels of HO‐1 and SOD in the liver were 50.63% and 82.36% higher than those of rats fed with HFD (*p* < .05), respectively. The relative gene expression levels of Nrf2, NQO1, CAT, SOD, and HO‐1 in rats fed with HFDM‐H were 84.22%, 180.32%, 54.65%, 118.38%, and 79.35% higher than those in rats fed with HFD (*p* < .05), respectively. In HFDP‐H rats, the expression levels of Nrf2, NQO1, CAT, SOD, and HO‐1 were 80.11%, 165.92%, 53.57%, 97.54%, and 65.83% higher than those in the HFD group (*p* < .05).

### 3.5 Antioxidant activity in vitro

The above results suggested that intact mung beans and peas can effectively improve oxidative stress injury induced by high‐fat diet in rats. However, the nutrients in these two kinds of legumes are complex, and it is difficult to determine which active ingredients play a key role in antioxidation. Therefore, in order to verify the main antioxidant components of mung beans and peas, these two kinds of legumes were extracted by sequential extraction methods, and the antioxidant activities of the extracts were studied in vitro and in cells. The antioxidant effects of water extracts were poor, and then, in this study, we focused on the antioxidant effects of ethanol extracts.

**Figure 5:**
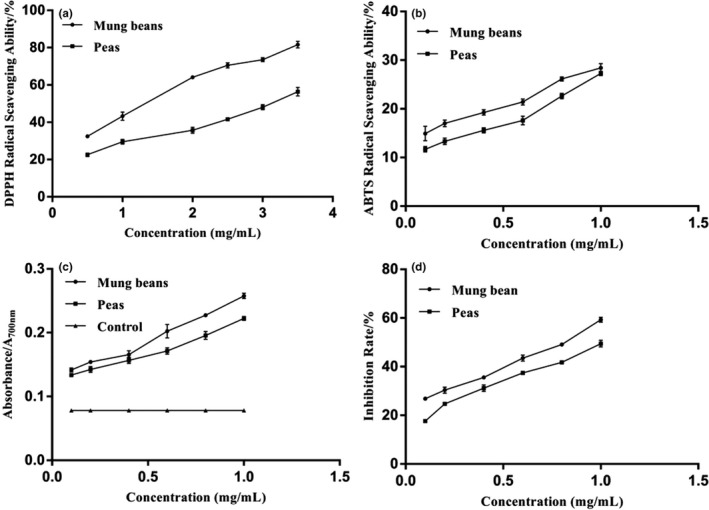
The antioxidant activities of mung beans and peas. (a) DPPH radical scavenging activity; (b) ABTS radical scavenging ability; (c) reducing power; and (d) antilipid peroxidation capacity

Ethanol extracts exhibited DPPH scavenging capacity as shown in Figure 5a. The DPPH scavenging capacity of mung bean and pea extracts increased with the increase in concentration. When the concentration of ethanol extracts was 1.0 mg/ml, the DPPH scavenging efficiencies of mung beans and peas were 80.81% and 56.58%, respectively. Figure 5b showed ABTS scavenging capacity of legume extracts. When the concentration of ethanol extracts was 1.0 mg/ml, the scavenging efficiencies of mung beans and peas were 28.22% and 27.25%, respectively. As shown in Figure 5c, the reducing power of the extracts increased with the increase in concentration. When the concentration of ethanol extracts was 1.0 mg/ml, the reducing power of mung beans and peas was 0.261 and 0.222, respectively. Figure 5d showed the results of antilipid peroxidation ability. When the concentration of ethanol extracts was 1.0 mg/ml, the antilipid peroxidation capacity of mung beans and peas was 58.82% and 49.76%, respectively. This result indicates that mung beans and peas have moderate antilipid peroxidation ability. With the increase in mung bean and pea concentrations, the antioxidant ability increased.

**Figure 6:**
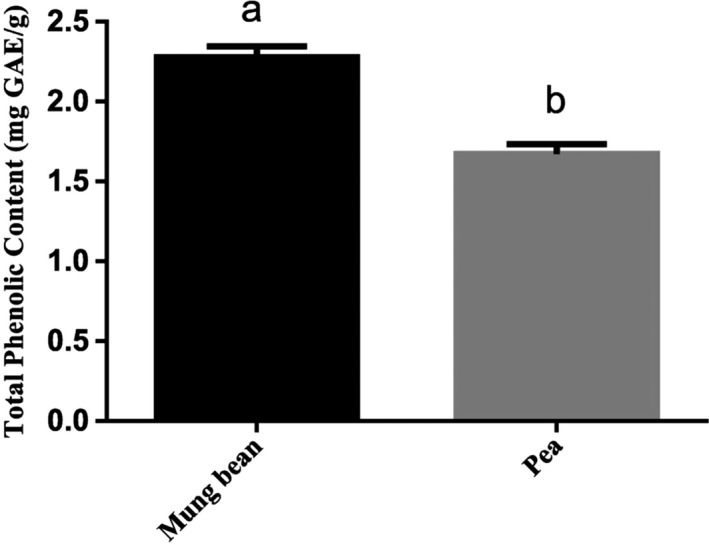
The total phenolic content of ethanol extracts. There were significant differences in the different expressions of superscripts (a, b), *p* < .05

As shown in Table 1, the contents of ferulic acid and protocatechuic acid in mung bean and pea showed significant difference, while the other components did not. The content of ferulic acid in mung bean and pea was 17.6 and 2.33 mg/100 g, and the content of protocatechuic acid was 400 and 11.1 mg/100 g, respectively. Figure 6 showed the total phenolic content of ethanol extracts from mung bean and pea, which are 2.28 and 1.67 mg GAE/g, respectively. It is reported that ferulic acid and protocatechuic acid are phenolic acids widely existing in plants, which have the functions of antioxidant, inhibiting tyrosinase activity and protecting nerves (Robbins, 2003). In general, the polyphenols and flavonoids in legumes are the main antioxidant components, and the content of phenols varies with the variety and origin of legumes.

### 3.6 Ethanol extract treatment in OA‐induced HepG2 cells

The mung bean ethanol extracts were not cytotoxic at every dose concentration (100, 200, and 400 μg/ml), whereas pea ethanol extracts were cytotoxic at 400 μg/ml (*p* < .05) (Figure 8a). Therefore, pea ethanol extracts at 100 and 200 μg/ml were used for the subsequent experiments.

**Figure 7:**
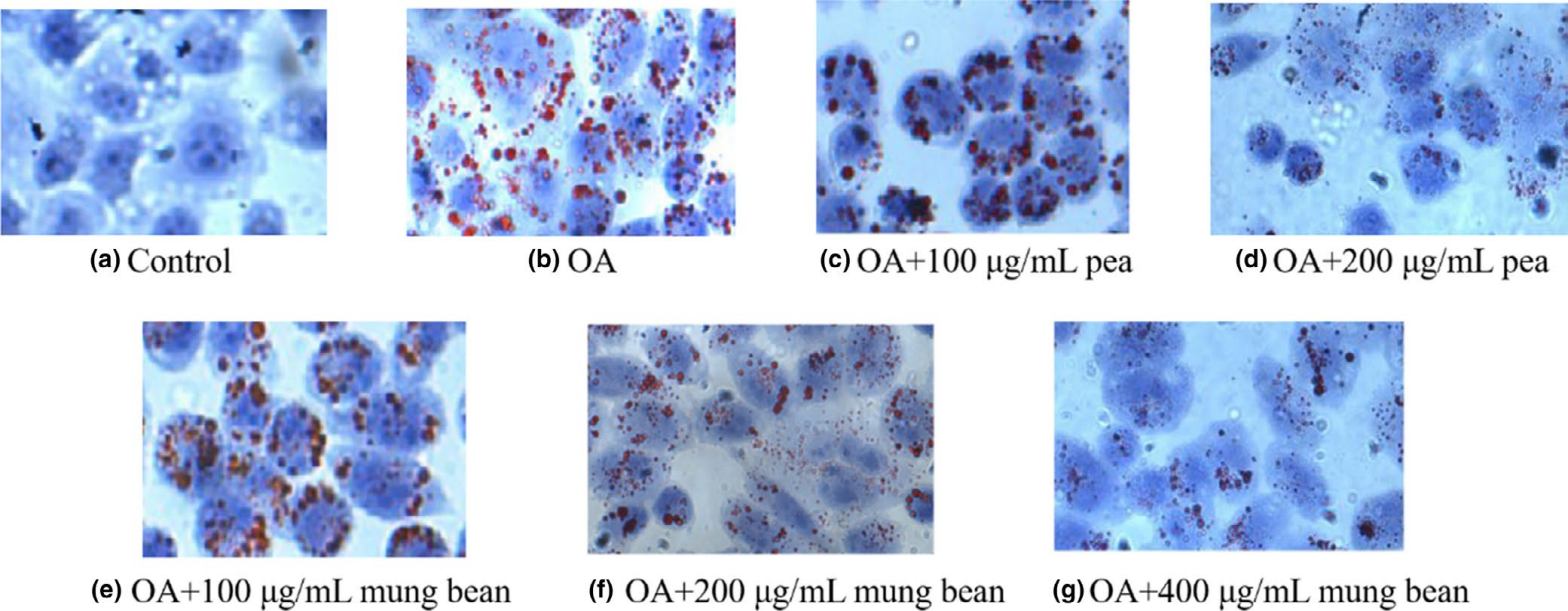
The Oil Red O staining of mung bean and pea ethanol extracts

**Figure 8:**
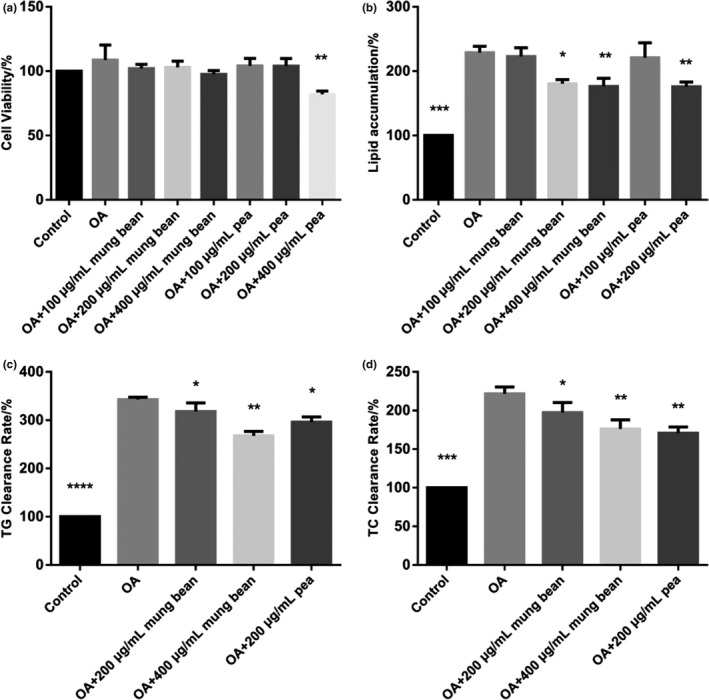
The cell viability, lipid accumulation and TG, and TC of mung bean and pea ethanol extracts. a: cell viability; b: lipid accumulation; c: TG level; and d: TC level

Oil Red O staining of HepG2 cells is shown in Figure 7. As shown in Figure 8b, compared with the control group, OA could obviously induce lipid accumulation. And co‐treatment with 1 mM OA and 200 and 400 μg/ml mung bean ethanol extracts and co‐treatment with 1 mM OA and 200 μg/ml pea ethanol extracts could significantly reduce the lipid accumulation induced by OA (*p* < .05). However, the concentration of ethanol extracts at 100 μg/ml reveals no significant difference compared with OA group. Thus, ethanol extracts at 200 and 400 μg/ml were used for all the subsequent experiments. The TG and TC levels were significantly reduced at 200 or 400 μg/ml of ethanol extracts (Figure 8c,d)

**Figure 9:**
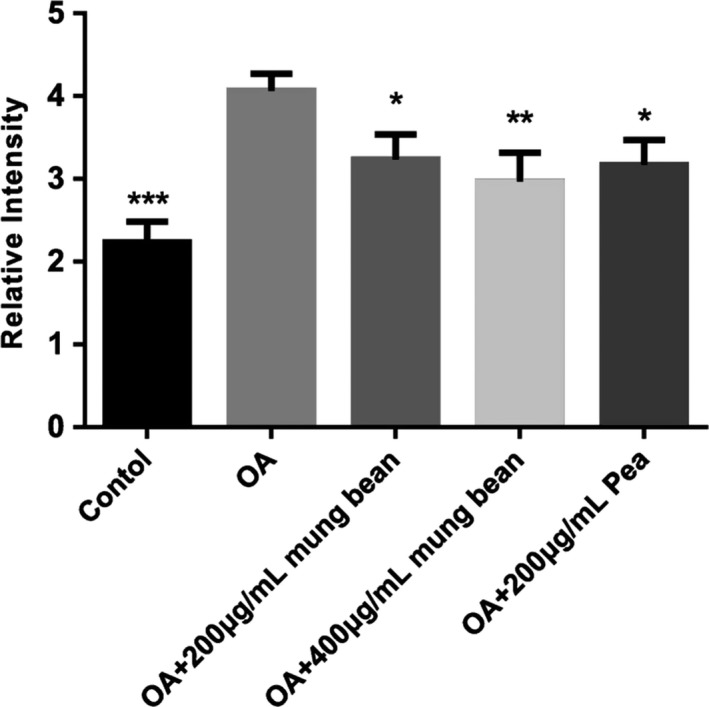
Effects of mung bean and pea extracts on ROS levels in HepG2 cells

**Figure 10:**
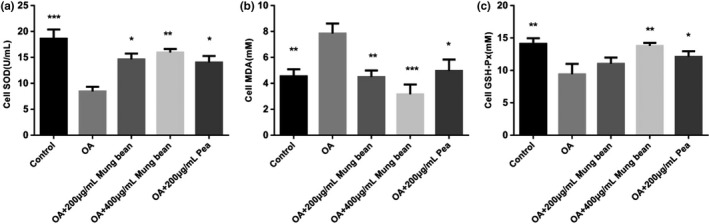
Effects of mung bean and pea extracts on antioxidant enzymes in HepG2 cells

Intracellular ROS levels were evaluated with the treatments of the mung bean and pea extracts on HepG2 cells. In the determination, the fluorescence intensity is correlated with the intracellular ROS generation. As shown in Figure 9, co‐treatment with 1 mM OA and 200 and 400 μg/ml mung bean ethanol extracts and co‐treatment with 1 mM OA and 200 μg/ml pea ethanol extracts caused marked decreases in ROS generation compared with OA group. Compared with OA group, ethanol extracts could markedly improve SOD, MDA, and GSH‐Px levels in cells (shown in Figure 10). Moreover, the ethanol extracts of mung beans with the concentration of 400 μg/ml had the best antioxidant effect.

## 4 DISCUSSION

Numerous studies showed that elevated serum levels of TC, LDL‐C, and TG could increase the incidence of atherosclerosis and cardiovascular disease. Conversely, elevated HDL‐C levels could reduce these risks (Assmann & Gotto, 2004). In this study, elevated serum TG and TC levels were observed in the HFD rats, which are consistent with previous reports (Xiao et al., 2010). However, the increases in LDL‐C and TC levels and decreases in HDL‐C could be significantly inhibited in the HFDM‐H rats, suggesting that mung beans and peas could be good candidates for blood lipid reducing. Yeap et al. (2015) found that fermented mung beans significantly reduced the serum levels of TC, TG, and LDL in mice compared with nonfermented mung beans. This might be for the reason that γ‐aminobutyric acid present in fermented mung bean may contribute to reducing the lipid in hypercholesterolemic mice. Abulnaja and Rabey (2015) found that the liver and heart tissues of rats fed with 2% cholesterol would lead to necrosis of hepatocytes and cytoplasmic blebbing. These findings observed in our study suggested that hypercholesterolemia was associated with pathological changes in vital organs.

Additions of mung beans and peas could protect liver and other tissues, and higher dose of legumes could almost return them to the normal states. Furthermore, high‐fat diet may induce the production of reactive oxygen species (ROS) (Wang, Shimada, Kato, Kusada, & Nagaoka, 2015). The biological effects of ROS were controlled by antioxidant enzyme defense mechanism in vivo. Supplementation with grain legumes could significantly enhance the activities of the main antioxidant enzymes in the serum and liver of HFD rats, and then improve the lipid oxidation induced by HFD. These findings were consistent with the researches in animals (Feillet et al., 2009) and humans (Patel et al., 2007), whose oxidative stress was induced by high‐fat and high‐carbohydrate diets. The activities of GSH‐Px, CAT, T‐AOC, and SOD, and the levels of MDA of the serum and livers of rats were used as indicators of oxidative stress after 4 weeks of treatment with different legumes. A daily consumption of mung bean and pea powders could enhance the liver antioxidant status as shown by the increased levels of GSH‐Px, CAT, and T‐AOC. No significant effects were observed in the activities of SOD and MDA under the same conditions. Different antioxidant enzymes react independently with different inducers (Kohen & Nyska, 2002). Some experiments have shown that dietary legumes are rich in polyphenols with high antioxidant capacity, which have an impact on the activities of antioxidant enzymes in vivo, such as increased SOD and GSH activities and decreased MDA levels (Kohen & Nyska, 2002). Nrf2, a key transcription factor in antioxidation, is widely distributed, especially in animal livers. The activities of CAT, SOD, NQO1, and HO‐1 increased with the increase in Nrf2 level (Vicente, Ishimoto, & Torres, 2014). Some natural antioxidants, including quercetin and blueberry, could enhance the mRNA expression of antioxidant enzymes by activating Nrf2 (Wang, Cheng, Zhang, Mu, & Wu, 2010). In this study, the expression levels of Nrf2, NQO1, CAT, SOD, and HO‐1 in the liver of adult rats fed with HFDM‐H or HFDP‐H were markedly higher than those of rats fed with HFD.

DPPH scavenging assays are usually used for the evaluation of the free radical scavenging of plant extracts for its simple sensitive and reproducible procedures. The plant source of extracts, environmental factors, and the solvent applied in the extractions may account for the differences in the levels of DPPH scavenging activities (Duenas et al., 2004). In this study, the DPPH scavenging capacity of mung bean ethanol extract was higher than the pea extract, which was agreed with the results of Cheng (2009). The higher DPPH scavenging capacity might be related to a higher content of natural antioxidants in mung beans, such as flavonoids (Nithiyanantham, Selvakumar, & Siddhuraju, 2012). ABTS radical scavenging activity of extracts is important and is exclusively measured by the ability of an antioxidant compound to be involved in a hydrogen atom transfer, which neutralizes generated ABTS^+^. The previous study showed that the ABTS scavenging ability of the pea methanol extract was 6,155.37 μmol/g (Xu & Chang, 2008). The total reducing power of legume extracts is also determined. Zhao, Du, Wang, and Cai (2014) found that the reducing power of undiluted ethanol extracted from mung bean was 0.96, and after diluting for five times higher, the reducing power of the raw mung bean extract was 0.23. Differences in the reducing power would be observed among the legume extracts, and the same results were also observed in this study. In addition, the absorbance of the reducing power determination increased with increasing extract concentrations. Many studies showed that phenolic compounds have strong antioxidant capacity (Randhir & Shetty, 2007). When reacting with oxide substances, these compounds can be used as hydrogen or electron donors. The total phenolic content of mung bean was reported to be 8.14 mg GAE/g (Yang, Cheng, Wang, Wang, & Ren, 2011), and 1.91 mg GAE/g for pea. Previous studies showed that the total flavonoid content of mung bean was 1.34 mg CE/g (Sreerama, Takahashi, & Yamaki, 2012) and that of pea was 1.10 mg CE/g. Furthermore, saponins in legumes also have antioxidant activities for their capacities of free radical capture (Kim et al., 2012). It should be pointed out that antioxidant substances, such as phenolic compounds, are mainly obtained by alcohol extraction, which might be one of the reasons for the strong antioxidant activity of legume ethanol extracts.

Most of the reported studies were focused on the extraction procedures or the antioxidant abilities in vitro. However, there are few reports about the effects of the ethanol extracts of legumes on oleic acid‐induced HepG2 cells which could act as a good model for studying extract antioxidant capacity, and the results could be easily quantified by an Oil Red O colorimetric technique. There are abundant phenolic compounds in legumes, especially for the large number of polyphenols. In this study, the results showed that the main antioxidant components might be present in the ethanol extracts. A proper ethanol extracts with oleic acids in a combination mixture might lead to significant lipoapoptosis and intracellular lipid accumulation with minimal cellular damage.

## 5 CONCLUSION

Results showed that after intact mung bean and pea supplementation, the antioxidant defense system and gene expression of rats were improved in varying degrees compared with HDF group. The water and ethanol extracts of mung beans and peas have strong DPPH scavenging activity, a moderate ABTS scavenging activity, and high antilipid peroxidation ability and reducing power. Meanwhile, the ethanol extracts of mung beans and peas in HepG2 cells have also strong antioxidant activities. These results suggested that mung beans and peas have potent antioxidant activity, but the specific components of the extracts were not clear. Therefore, further research and discussion were needed. And this study is also worthy of further research and development in food and pharmaceutical industries.

## CONFLICTS OF INTEREST

The authors declare no conflict of interest.

## ETHICAL STATEMENTS

The authors declare that there is no conflict of interest regarding the publication of this article. The research described herein was performed on male SD rats. This study was performed in strict accordance with protocols approved by Institutional Animal Care and Use Committee of University of Shanghai for Science and Technology, Shanghai, China.
